# Urban places and Mental Health challenges (lessons learned from Covid-19 crisis)

**DOI:** 10.1093/eurpub/ckac129.706

**Published:** 2022-10-25

**Authors:** M Jevtic, V Matkovic, MP Kusturica, C Bouland

**Affiliations:** 1 Faculty of Medicine, University of Novi Sad, Novi Sad, Serbia; 2 Institute of Public Health of Vojvodina, Novi Sad, Serbia; 3 School of Public Health, Université Libre de Bruxelles, Brussels, Belgium; 4 Health & Environment Alliance, Brussels, Belgium

## Abstract

Nature deprivation under COVID-19 lead to reduced well-being. Urban design interventions were also identified as an important contributor to the restoration of community confidence, choice, and safety. Factors related to sociodemographic, housing and lockdown were linked to changes in exposure to nature during the pandemic lockdown. Changes in exposure to nature and mental health outcomes during the COVID-19 lockdown were strongly linked. Especially young people had an increased number of mental health problems. Children and youth were more bored and worried in comparison with the pre-pandemic period. The educational institutions worldwide were closed or changed to online education during the pandemic, leading to great disturbance in students’ education and outdoor events. All “green infrastructure” (GI) resources (including parks, gardens etc.) received great attention as “essential infrastructure” supporting well-being. But, the quality, functionality and position of GI in urban areas showed inequality in distribution. Frequently, societies with greater ethnic diversity, lower income and larger health inequality suffered from unsatisfactory or lack of access. GI is important in decision-making to address inequality. This work will also present an open-air activity for all generations: A reflective walk through the oldest part of Novi Sad (EU Capital of Culture 2022), as a part of Project Reflective citizens in Novi Sad. This walking tour was led by pupils from primary school - where all generations spend useful time in open space and a safe atmosphere walking tour, learning and listening about the cultural history of the oldest part of the city. It is vital to enhance urban planning and design practices in making healthier and more resilient communities. It is necessary to underline the importance of planning green spaces that need time to form in urban areas, and which have proven to be very important for mental health in the midst of the pandemic crisis.

